# Evaluating the use of the EORTC patient-reported outcome measures for improving inter-rater reliability of CTCAE ratings in a mixed population of cancer patients: study protocol for a randomized controlled trial

**DOI:** 10.1186/s13063-020-04745-w

**Published:** 2020-10-13

**Authors:** Lisa M. Wintner, Johannes M. Giesinger, Monika Sztankay, Andrew Bottomley, Bernhard Holzner, on behalf of the EORTC Quality of Life Group

**Affiliations:** 1 Department of Psychiatry, Psychotherapy and Psychosomatics, University Hospital of Psychiatry I, Medical University of Innsbruck, Anichstr. 35, 6020 Innsbruck, Austria; 2 European Organisation for Research and Treatment of Cancer (EORTC Headquarters), Avenue E. Mounier 83/11, 1200 Brussels, Belgium

**Keywords:** Patient-reported outcomes (PROs), Common Terminology Criteria for Adverse Events (CTCAE), Inter-rater reliability, Electronic data assessment, Cancer

## Abstract

**Background:**

In oncology, detection and tracking of adverse events are of top priority and rely mostly on the Common Terminology Criteria for Adverse Events (CTCAE). Besides, clinical trials use as well patient-reported outcomes (PROs) to assess those adverse events, which are only accessible through patient self-reporting, such as fatigue, pain, and sleep disorders. Especially those issues that are not visible from the outside are often misinterpreted and underestimated by mere provider ratings. This trial aims at evaluating the impact of providing PRO data to providers on the accuracy of adverse event assessment in terms of inter-rater reliability of CTCAE ratings.

**Methods:**

The trial uses a cross-sectional, unblinded, randomized controlled trial design with two trial arms and a single assessment time point. Eligible patients (aged 18 and above, any cancer diagnosis, currently under treatment, inpatient or day clinic setting, present symptom burden, no psychiatric or mental problems, written informed consent) complete an electronic version of the EORTC QLQ-C30 and 16 additional questions taken from the EORTC Item Library. PRO data is immediately processed and made available to CTCAE rating providers for conducting their ratings during the medical encounter. Patients are randomly assigned 1:1 to the intervention group (providers see PRO results on the same screen as the CTCAE rating) and the control group (no access to PRO data during the CTCAE rating). A superiority analysis will compare the inter-rater reliability (using intra-class correlation (ICC) coefficients) between the control and the intervention groups for each adverse event evaluated.

**Discussion:**

The presented trial will demonstrate potential benefits of using PRO measures to improve the reliability of CTCAE ratings in cancer trials and the identification of adverse events. The new insights gained may lead to a new strategy for evaluating adverse events in clinical trials by combining patient and provider ratings. This might also have implications for daily clinical practice and cancer registries.

**Trial registration:**

ClinicalTrials.gov NCT04066868. Registered on August 26, 2019. Competence Center for Clinical Trials of the Medical University of Innsbruck 20190513-2007. Registered on May 14, 2019. (version 6.0, March 18, 2019)

## Background

In oncology, the Common Terminology Criteria for Adverse Events (CTCAE) (developed by the US-American National Cancer Institute (NCI) of the National Institute of Health (NIH) [1]) are an established method to evaluate and document the toxicities in clinical studies. CTCAE are a provider-based (clinicians or nurses) grading system, which facilitates the classification of adverse events (AEs) regarding their severity from mild to life-threatening to event-related death. During the last decades, the growing awareness of the importance to complement the traditional provider-rated assessment of the patient’s health status by patient-reported outcomes (PROs) triggered the development of valid and reliable instruments [2]. As “a measurement of any aspect of a patient’s health status that comes directly from the patient” [3] without being interpreted or altered by anyone else, PRO represents the gold standard to capture the patient’s perspective regarding his/her health status. PRO covers a wide range of both complex concepts such the patient’s functioning (physical, social, emotional, cognitive), depression, or anxiety as well as specific symptoms and overlaps strongly with CTCAE, especially with regard to the latter. Though the parallel collection of CTCAE and PRO ratings reflects a common practice in cancer clinical trials, their combination in order to maximize information yield is still uncharted territory.

Several studies found substantial discrepancies when it comes to comparisons of AE ratings from patients with those from providers. For non-observable symptoms such as fatigue or dyspnea, concordance of patient and clinician ratings was worse than for observable symptoms such as vomiting and diarrhea [4] and patients rated symptoms like fatigue, dyspnea, or skin toxicities more frequently and more severely than providers did [5–7]. Clinician ratings also lack sensitivity [2] and underestimate the severity of AEs [4]. A review of the literature on direct comparisons of PRO and CTCAE also reports that predominantly poor to moderate associations between clinical and patient-based AEs can be observed [8]. Thus, especially for those AEs, which can only be assessed via patients’ self-reports, the integration of PROs to collect information on AEs is a worthwhile strategy to improve this situation.

Besides the lacking concordance of patient-based and provider-based AE ratings, it is notable that even providers often achieve only moderate agreement. The CTCAE system itself has not undergone a formal validation procedure [5], and to the best of our knowledge, only two studies have investigated reliability of CTCAE ratings and reported only moderate rater agreement [9, 10].

Considering the abovementioned findings, integrating patient assessment with PRO measures into a multi-method strategy for AE identification may result in a substantial improvement of clinical trial methodology. To date, PRO and CTCAE ratings have been used in parallel rather than as a combined source for drug safety information. Integrating PRO data providing standardized information on toxicities into the process of CTCAE rating may increase inter-rater reliability as well as AE identification, in particular with regard to low-grade toxicity that is not related to salient clinical events such as unplanned hospitalization.

## Methods

### Trial objectives and endpoints

The trial aims at evaluating the impact of providing PRO data to providers on the accuracy of AE information, assessed by using CTCAE ratings. As there is no gold standard for AE ratings available, accuracy will be investigated in terms of inter-rater reliability.

The primary trial endpoint is the inter-rater reliability of CTCAE provider ratings for 17 AEs in cancer patients with mixed diagnoses. A superiority analysis will compare the inter-rater reliability between the control and the intervention groups for each assessed AE.

There are two secondary trial endpoints: (1) difference in the frequency of identified AEs (any grade) between the intervention and control group and (2) the comparison of the differences in inter-rater reliability between the intervention and control group across the different types of AEs.

The selection of CTCAE toxicities is based on the AEs detected by the European Organization for Research and Treatment of Cancer Quality of Life Core Questionnaire (EORTC QLQ-C30), which cover symptoms normally associated with chemotherapeutic treatments. In addition, AEs associated with immunotherapy have been added as this treatment option is becoming increasingly common in various diagnoses. The following 17 CTCAE toxicities will be assessed in this trial: anxiety, depression, irritability, concentration impairment, memory impairment, fatigue, pain, dyspnea, nausea, vomiting, insomnia, anorexia, diarrhea, constipation, peripheral sensory neuropathy, rash, and pruritus.

### Trial design

The trial objectives will be investigated using a cross-sectional, unblinded, randomized controlled trial design with two trial arms and a single assessment time point.

### Trial setting

The Medical University of Innsbruck acts as the trial sponsor. The trial will be conducted at six different sites across Europe and Asia: County Hospital of Kufstein (Kufstein, AT), Besançon University Hospital (Besançon, FR), Martin-Luther-University Halle-Wittenberg (Halle, DE), University of Cagliari (Cagliari, IT), General King Hussein Cancer Centre (Amman, JOR), and Kansai Medical University Hospital (Osaka, JP). The trial will recruit inpatients or those attending oncology day care. Patient recruitment is planned for a duration of 27 months.

### Trial participants

To be eligible for inclusion in the trial, patients have to fulfill the following inclusion criteria:

- Patients aged 18 or above
- Any cancer diagnosis (no more than 20% per diagnostic group)
- Current treatment with chemotherapy or immunotherapy
- Inpatient or day clinic setting
- Scoring 3 or above on an initial screening question (“On a scale from 0 to 10, to what degree did you experience physical or emotional symptoms/problems during the last week?”)
- No psychiatric or mental problems (i.e., no such diagnosis in the medical records)
- Written informed consent

Participating providers are requested to be either a medical, surgical, or radiation oncologist by training or a specially trained nurse authorized to perform CTCAE assessments in clinical trials, both with at least 1-year experience in oncology.

### Withdrawal

The only criterion for trial dropout is the participant’s wish to do so. This applies to both providers and patients. Withdrawal is possible at any time and does not result in any negative consequences for the former participant.

### Trial procedure

**Fig. 1:**
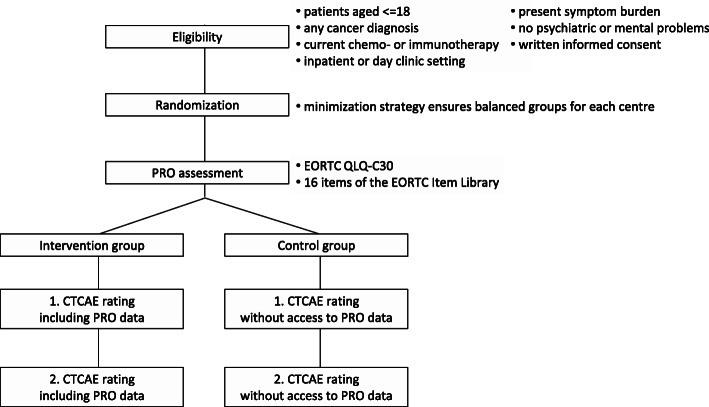
Flow chart of the trial procedure

**Fig. 2:**
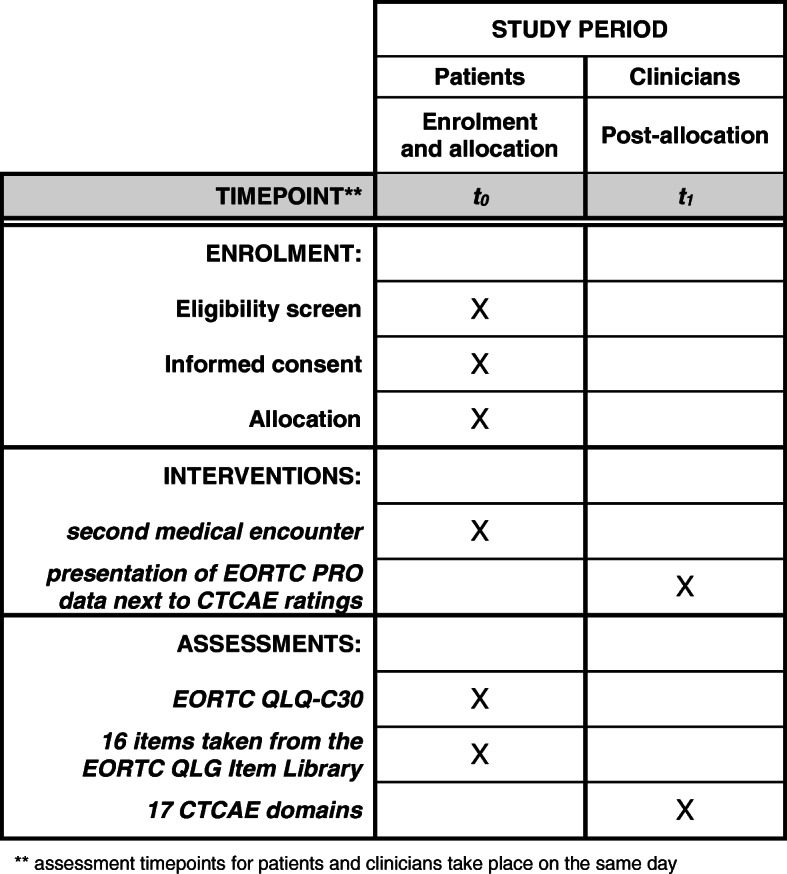
Standard Protocol Items: Recommendations for Interventional Trials (SPIRIT) figure

During their day clinic or inpatient stay, all patients provide PRO data by autonomously completing questionnaires (EORTC QLQ-C30 and additional items from the EORTC Item Library) on a tablet PC. Subsequently, after having a medical encounter with the patient, two providers conduct consecutive, independent CTCAE ratings (intervention group: including PRO data, control group: no additional information). All data will be collected on the same day. Figure 1 depicts the trial flow, and Fig. 2 shows the Standard Protocol Items Recommendations for Interventional Trials (SPIRIT) figure [11].

The software Computer-based Health Evaluation System (CHES [12]) is used for electronic PRO questionnaire completion, collection of CTCAE ratings, and completion of electronic CRFs. If it is necessary to assess PROs with paper questionnaires, the questionnaires will be entered into the system right after completion into the trial database to allow in the intervention group electronic result presentation to the provider. Each provider has an individual login, granting access to CTCAE ratings and preventing double ratings by the same provider. Login to the software is possible via any computer or mobile device with an Internet connection. Providers receive the instruction to open the CHES software upfront the patient encounter and to complete the CTCAE rating during the consultation.

Allocation of providers to patient CTCAE ratings does not follow a predefined rule. Providers on duty are requested to complete CTCAE rating on the same day the patient has completed PRO data. If during medical consultations the need for additional treatment or side-effect management becomes apparent, the provider in charge will provide medical advice or referral to other specialists.

### Intervention

In the *control group*, providers use the assessment software to access and complete the CTCAE rating in an electronic format.

**Fig. 3:**
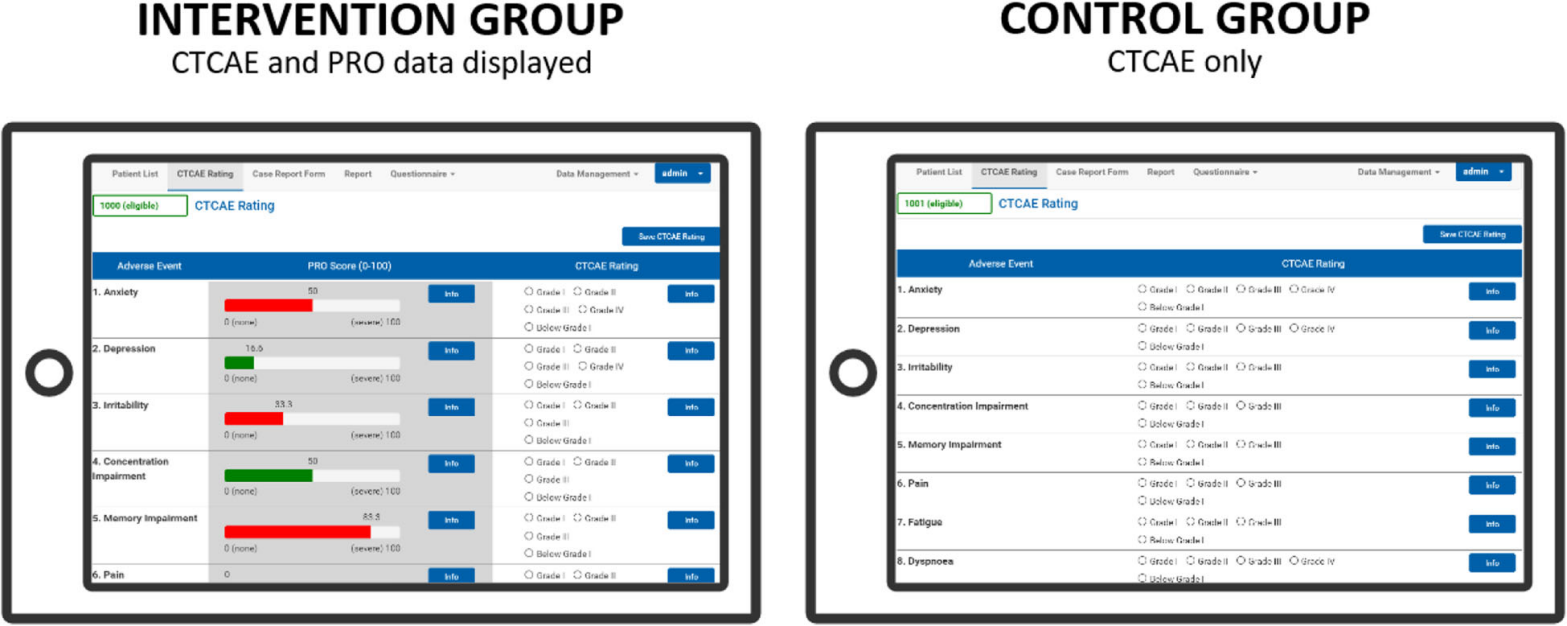
Presentation of the graphical design of CTCAE ratings and PRO data according to the allocated trial group

In the *intervention group*, providers see the respective patient’s PRO data next to the corresponding CTCAE domain, when accessing the rating. For an example of the CTCAE rating with and without displayed PRO data, see Fig. 3. In addition, providers can access the individual PRO questions and answers. This is especially important for subscales of the EORTC QLQ-C30, which comprise different CTCAE categories (e.g., the questions of the scale EORTC Emotional Functioning include the single CTCAE categories depression, anxiety, and irritability).

Thus, the intervention is the additional provision of PRO data to providers who complete a CTCAE rating for the respective patient. To allow for a naturalistic setting, providers do not receive any special training in CTCAE ratings.

### Randomization and blinding

The PRO assessment software processes a 1:1 randomization to the control or intervention group for each study center. In order to ensure balance between the two groups, minimization is applied when the patient’s basic data is added to the database. For this, a weighted *cutoff* is determined taking the sizes of control and intervention groups into account. Next, a random number between 0.0 and 1.0 is generated. If the random number is smaller than the *cutoff*, the patient is added to the group with less patients. In order to determine *weight*, the randomization procedure has been simulated and *weight* = 0.05 has been determined to produce the desired trade-off between balancing the two groups and preventing predictable group assignments. For each patient, a detailed randomization log is produced, which allows tracing all steps of the randomization procedure.

Providers cannot be blinded in this trial. At the time of PRO assessment, patients do not know whether they are assigned to the study or the control group.

### Assessment instruments

Collection of AE ratings is done for those CTCAE toxicities that are also covered in the EORTC QLQ-C30 and AEs, which are common in patients receiving immunotherapy (peripheral sensory neuropathy, rash, and pruritus).

### Case report form

The case report form for patients contains an eligibility checklist (cancer diagnosis, age at diagnosis above 18 or above, current chemotherapy or immunotherapy, ability to understand the questionnaire cognitively and linguistically, written informed consent provided, no psychiatric or mental problems) and a data sheet for clinical and sociodemographic data (sex, age, country of origin, marital status, living situation, education level, employment status, date of diagnosis, diagnosis, metastasis, disease stage, recurrence, current treatment and treatment intention, concurrent medication, comorbidities, performance status). For patients who discontinue or refuse to participate in the study, the reasons for their decision, sex, age, and diagnosis are documented.

For providers, age, sex, specialty, years of professional experience, previous participation in clinical trials, and research experience (self-reported years of experience) are collected on a separate sheet.

### PRO assessment

Patients complete a total of 46 questions covering 17 adverse events listed in the CTCAE. The EORTC QLQ-C30 by the European Organization for Research and Treatment of Cancer (EORTC) Quality of Life Group [13] was originally developed for use in clinical trials, covers physical and psychosocial aspects, and is one of the most widely used instruments in Europe. It consists of 30 items assessing 5 functioning scales (physical, social, role, emotional, and cognitive functioning), 3 symptom scales (fatigue, pain, and nausea/vomiting), 6 single-item symptoms (appetite loss, dyspnea, diarrhea, constipation, sleeping difficulties, financial difficulties), and a Global Health and Quality of Life scale. In addition, 16 items have been taken from the EORTC Item Library to supplement those CTCAE domains which are covered by one question in the EORTC QLQ-C30 and to include AEs common in chemotherapy and immunotherapy (peripheral sensory neuropathy, rash, and pruritus).

### CTCAE

The NCI has published standardized definitions for AEs, known as the Common Terminology Criteria for Adverse Events (CTCAE), to describe the severity of organ toxicity for patients receiving cancer therapy. The most recent CTCAE version 5.0 was published in November 2017 and became effective in April 2018 [1].

In this trial, providers rate the following 17 CTCAE toxicities: anxiety, depression, irritability, concentration impairment, memory impairment, fatigue, pain, dyspnea, nausea, vomiting, insomnia, anorexia, diarrhea, constipation, peripheral sensory neuropathy, rash, and pruritus.

For the CTCAE ratings, the electronic form includes an info box that shows the exact definitions for each grade as given in the CTCAE version 5.0. Depending on the respective domain, rating from grade I to III or IV is possible. An additional “below grade I” category allows the provider to report that a certain problem is not present (or not qualifying for grade I or above).

### Statistical analysis

Only complete data sets will be used for final analysis. For analysis of the primary trial endpoint, we will compare inter-rater reliability of CTCAE ratings separately for each domain using intra-class correlation (ICC) coefficients. We would like to note that difference in ICC is an unusual endpoint, and to the best of our knowledge, no recommendations are available from the literature what constitutes a minimal important difference in ICC. According to Cicchetti [14], ICCs can be classified as follows: < 0.40 poor, 0.40–0.59 fair, 0.60–0.74 good, and > 0.75 excellent. This is consistent with the classification by Fleiss et al. [15].

For the analysis of the secondary trial endpoints, ICCs will also be compared across AEs to evaluate differences in the reliability of the CTCAE ratings across different types of AEs (e.g., AEs differing in observability). In addition to intra-class correlations, we will also compare percentage of absolute agreement of ratings and percentage for deviations by one and two grades. The frequency of identified AEs (any grade) will be compared between the intervention and control group using Fisher exact tests.

### Power analysis

A sample size of 1024 patients (512 per group) allows to detect differences in ICC with an effect size of *q* = 0.174 (alpha = 0.05, beta = 0.20, two-sided). This effect size corresponds to differences in reliability of, e.g., 0.20 vs 0.36, 0.40 vs 0.54, or 0.60 vs 0.70. Power analysis for ICC has been approximated by Pearson correlation coefficients as suggested by Streiner et al. [16]. The planned sample size is sufficient to detect a difference corresponding to about half a category width in this classification.

### Data security, data management, and data monitoring

Data is stored securely on an EORTC server. Patient data is entered and stored pseudonymized, i.e., that patients receive an ID from the respective study center, which makes it impossible to determine the identity of the individual patient within the system. An ID list is kept at each participating center, with which de-pseudonymization can be carried out; this list remains at the center and is stored there in accordance with data protection regulations.

Each collaborating center will perform data quality management by conducting plausibility checks (e.g., checking minimum and maximum values) and identifying missing values. Final data analysis will be conducted at the institution of the principal investigator.

A data safety monitoring board is not considered necessary as the study does not include procedures, medications, or interventions that expose patients to a risk of potential harm. Conducting safety audits would possibly cause more inconvenience to patients than it would protect them from possible risks, which are practically not to be expected when filling out a questionnaire and conducting a medical interview. In accordance with the rules of the Project and Module Development Committee of the EORTC Quality of Life Group, an interim scientific report on the progress of the study will be prepared before each of the six-monthly meetings of the EORTC Quality of Life Group in order to monitor recruitment rates and identify areas for improvement.

## Discussion

Providing evidence for improved CTCAE ratings in cancer trials may have a substantial impact on how PROs are used in future trials and further strengthen the perceived relevance of the patients’ perspective (operationalized by using PRO measures) for outcome and safety assessment in cancer research. This may result in a new AE assessment strategy making combined PRO and provider ratings the new standard method. Naturally, enhanced AE assessment is beneficial not only in the context of clinical trials, but also with regard to daily oncological practice, cancer registries, and pharmacovigilance.

### Trial status

At the time of submission of the protocol (version 6.0, March 18, 2019) for publication, two centers were collecting data, recruitment started in early February 2020, three were in the final phase of study set-up, and one had to postpone patient recruitment until autumn 2020 due to the COVID-19 pandemic. According to the recruitment phase of about 27 months, recruitment will continue until about early 2022 or until the required number of patients is enrolled in the study.

## Data Availability

The data gathered by conducting this trial may be made available by the EORTC (https://www.eortc.org/request-for-data/) upon reasoned request.

## Abbreviations

AE: Adverse event
CTCAE: Common Terminology Criteria for Adverse Events
EORTC: European Organization for Research and Treatment of Cancer
EORTC QLQ: European Organization for Research and Treatment of Cancer Quality of Life Questionnaire
EORTC QLQ-C30: European Organization for Research and Treatment of Cancer Quality of Life Questionnaire Core 30
PRO: Patient-reported outcome
ICC: Intra-class correlation
